# Non-motor symptoms in multiple system atrophy: A comparative study with Parkinson's disease and progressive supranuclear palsy

**DOI:** 10.3389/fneur.2022.1081219

**Published:** 2023-01-23

**Authors:** Wen-Zheng Hu, Ling-Xiao Cao, Jin-Hui Yin, Xue-Song Zhao, Ying-Shan Piao, Wei-Hong Gu, Jing-Hong Ma, Zhi-Rong Wan, Yue Huang

**Affiliations:** ^1^China National Clinical Research Center for Neurological Diseases, Beijing Tiantan Hospital, Capital Medical University, Beijing, China; ^2^Department of Neurology, Beijing Tiantan Hospital, Capital Medical University, Beijing, China; ^3^Traditional Chinese Medical Department, Beijing Tiantan Hospital, Capital Medical University, Beijing, China; ^4^Neurology Department, China-Japan Friendship Hospital, Beijing, China; ^5^Neurology Department, XuanWu Hospital, Capital Medical University, Beijing, China; ^6^Department of Neurology, Aerospace Central Hospital, Beijing, China; ^7^Department of Pharmacology, Faculty of Medicine and Health, School of Medical Sciences, University of New South Wales, Sydney, NSW, Australia

**Keywords:** progressive supranuclear palsy, Parkinson's disease, disease progression, non-motor symptom, multiple system atrophy

## Abstract

**Background:**

Non-motor symptoms (NMS) are compulsory clinical features for the clinical diagnosis of multiple system atrophy (MSA), some of which precede motor symptoms onset. To date, few studies have systematically investigated NMS in MSA and the timing of presenting NMS as the disease progresses. Clinically, MSA is difficult to be differentiated from Parkinson's disease (PD) and progressive supranuclear palsy (PSP), and the differences in NMS between MSA and PD/PSP remain unclear. The aim of this study was to compare the burden of NMS between MSA and PD/PSP and to delineate the timing of NMS presentation relative to the onset of motor symptoms in MSA.

**Methods:**

A total of 61, 87, and 30 patients with MSA, PD, and PSP, respectively, were enrolled in this study. NMS was systematically assessed in all patients using the NMS scale (NMSS), and the onset of NMS relative to the onset of motor symptoms in MSA was investigated.

**Results:**

MSA group had higher total NMSS scores (82.15 ± 46.10) than the PD (36.14 ± 30.78) and PSP (50.30 ± 55.05) groups (*p* < 0.001 overall). The number distribution pattern of the NMS was significantly different among the three parkinsonian disorders (*p* < 0.001 overall). In total, 85.2% of patients with MSA had more than 10 NMS, which was significantly higher than PD (28.7%) and PSP (33.3%). The frequency and scores of many NMSS subdomains and symptoms were higher in MSA than in PD and PSP (all *p* < 0.05). Multivariate logistic regression analysis revealed that patients with fainting, lack of motivation, swallowing, and loss of sexual interest could be attributed to MSA rather than PD or PSP, while patients with loss of concentration and forgetfulness were characteristic features of PD or PSP rather than MSA. REM-sleep behavior disorder (RBD), constipation, problems having sex, and loss of sexual interest preceded the motor symptoms onset of MSA by 2.81 ± 4.51, 1.54 ± 6.32, 1.35 ± 4.70, and 0.45 ± 3.61 years, respectively.

**Conclusion:**

The NMS spectrum in MSA differs from that of PD and PSP. Patients with MSA have a higher NMS burden than patients with PD or PSP. RBD, constipation, problems having sex, and loss of sexual interest may become early diagnostic clinical markers of MSA.

## Introduction

Multiple system atrophy (MSA) is generally considered a sporadic adult-onset neurodegenerative disease characterized by a clinical combination of parkinsonism, autonomic, cerebellar, or pyramidal symptoms and signs (1, 2). It has been classified into two subtypes, namely, MSA with predominant cerebellar ataxia (MSA-C) and MSA with predominant parkinsonism (MSA-P) (3). Parkinson's disease (PD) and progressive supranuclear palsy (PSP) are two other neurodegenerative disorders that share parkinsonian clinical features with MSA (4). Due to overlapping motor symptoms, the differential diagnosis of MSA from PD and PSP is difficult, especially at the early stages of the disease (5).

Non-motor symptoms (NMS) are common clinical features of MSA, PD, and PSP (6–9) and have been identified as one of the most important predictors of quality of life (QoL). In PD, many studies have investigated the NMS individually or systematically (8, 10–12). While in MSA and PSP, NMS is still poorly investigated in clinical practice and remains undeclared. Some studies have investigated a few individual NMS in MSA (13–15), but few studies have systematically investigated NMS of MSA (16, 17), so does the studies on PSP (18–20). Previous studies have compared individual NMS between MSA, PD, and PSP (21–27) and found that NMS may be useful in the differential diagnosis between MSA and PD/PSP. In the urinary system, urinary urgency and increased frequency are frequently reported in patients with PD and MSA (28, 29); however, the prevalence and magnitude of these symptoms in patients with MSA are typically greater than those in patients with PD (25, 26). In the cardiovascular system, the presence of orthostatic hypotension distinguishes MSA from PSP, particularly in the early stages (30, 31). In terms of cognitive function, patients with PSP show more severe cognitive impairment than patients with PD and MSA (22). RBD is significantly more common in MSA when compared with PD and PSP patients (27). To the best of our knowledge, few studies have systematically assessed NMS among these three parkinsonian disorders. Lee et al. (32) have compared NMS in MSA, PD, and PSP by using the Non-Motor Symptom Scale (NMSS), but have not compared the frequency of NMS, so the differences in the prevalence of NMS among MSA, PD, and PSP remain unclear.

Some NMS, including hyposmia, REM sleep behavior disorder (RBD), constipation, and depression, may precede the onset of motor symptoms in PD (33–36). Regardless of the predominant motor features, the MSA clinical spectrum also includes various non-motor symptoms that may precede the onset of motor symptoms (37). A previous study has shown that NMS precedes the onset of motor symptoms in 23.0% of patients with MSA (38). In addition, a group of studies has reported that autonomic symptoms were the first manifestations in 31.8–73.0% of patients with MSA (39–43). RBD, erectile failure, postural lightheadedness, and stridor are frequently observed in MSA populations, and these symptoms have been reported to precede the onset of motor symptoms. To date, 12 studies have reported RBD preceding the clinical MSA onset, and the prevalence of RBD as the first symptom of disease onset ranged from 10.0 to 60.0% when calculated in overall MSA cases (44). A large monocentric study on 158 patients with MSA shows that RBD was the most frequent first symptom of MSA, preceding the disease onset by a median of 3 (2–5) years (40). While another retrospective study of 30 patients with MSA showed that RBD was the third most frequent initial symptom of MSA, after complete erectile failure and postural lightheadedness (43). In addition, studies have shown that erectile failure is the first autonomic symptom in 80.0% of men with MSA (40) and stridor is the initial symptom in 4.0–5.2% of patients with MSA (39, 45). A previous clinicopathological study found symptomatic orthostatic hypotension occurred within the first year after disease onset in MSA, and the latency to onset of symptomatic orthostatic hypotension was significantly shorter than that of PD (31). A recent review focused on NMS in MSA, detailing different domains and timing of onset in MSA, PD, and other parkinsonism disorders (37). As mentioned above, although many studies have reported that some NMS precede the onset of motor symptoms in MSA, the timing of other NMS presentations relative to motor symptom onset remains undeclared.

The aim of this study was to explore the profile of NMS in patients with MSA using NMSS and to compare NMS in patients with MSA, PD, and PSP. Furthermore, the timing of NMS presentation relative to the onset of motor symptoms in MSA and the role of NMS in the diagnosis of prodromal MSA were investigated.

## Materials and methods

### Study subjects

This study was conducted at the China National Clinical Research Center for Neurological Diseases (NCRCND), Beijing Tiantan Hospital, Capital Medical University, between September 2020 and May 2022. Patients were recruited continuously through physician referrals from the Neurology Department, Beijing Tiantan Hospital, and the other three hospitals in Beijing (Xuanwu Hospital, China-Japan Friendship Hospital, and Aerospace Central Hospital), and their diagnoses were further confirmed by a neurologist at NCRCND. In the end, 61 MSA, 87 PD, and 30 PSP with the consensus of clinical diagnosis by at least two neurologists and completed comprehensive clinical tests were enrolled in this study. Possible or probable patients with MSA were diagnosed according to the second consensus statement on the diagnosis of MSA by Gilman et al. (46). Patients with MSA who met the above diagnostic criteria were re-checked through the latest 2022 version of the MSA diagnostic criteria and met the criteria for clinically established and clinically probable MSA (47). The patients with PD were diagnosed according to MDS clinical diagnostic criteria for Parkinson's disease (48). The patients with PSP were diagnosed according to the clinical diagnostic criteria for PSP (49). Subjects with concomitant cardiovascular, prostatic, or gastrointestinal diseases that could be associated with any of the NMS assessed were excluded. All subjects enrolled in the study provided written informed consent. This study was approved by the Institutional Review Board/Ethics Committee of Beijing Tiantan Hospital, Capital Medical University, Beijing, and was conducted in accordance with the Declaration of Helsinki.

### Clinical assessments

Demographic details such as sex, age, age at onset, height, weight, duration of illness, and education were recorded. Disease onset was defined as the initial presentation of any motor symptoms. The disease severity was rated by the Unified Multiple System Atrophy Rating Scale, Part IV (UMSARS-IV) for MSA, and by the modified Hoehn & Yahr stage (H&Y stage) for PD and PSP. The motor symptom was assessed by the Unified Multiple System Atrophy Rating Scale, Part II (UMSARS-II) for MSA and by the Movement Disorders Society–revised Unified Parkinson's Disease Rating Scale, Part III (MDS-UPDRS-III) for PD and PSP. All participants underwent Mini-Mental State Examination (MMSE) task for the assessment of global cognitive function, the Pittsburgh Sleep Quality Index (PSQI) questionnaire for the assessment of sleep quality, the 24-item Hamilton Depression Scale (HAMD) assessment for measuring depressive symptoms, and the 14-item Hamilton Anxiety Scale (HAMA) task for the assessment of anxiety symptoms. RBD in MSA was assessed by the RBD Screening Questionnaire (RBDSQ).

The NMS was assessed by the NMSS. This scale consists of 30 items, covering nine subdomains: cardiovascular, sleep/fatigue, mood/apathy, perceptual problems/hallucinations, attention/memory, gastrointestinal, urinary, sexual function, and miscellaneous. The severity (0–3), frequency (1–4), and final score (severity × frequency) of each item are evaluated separately, and the total score for a subdomain is obtained by the sum of individual item scores. The total NMSS score ranges from 0 to 320, and higher scores are indicative of higher severity and frequency of NMS (50, 51).

The timing and latency of NMS on the NMSS scale from the onset of motor symptoms were recorded in the MSA group. The timing and latency of the RBD, as well as urinary incontinence (two important NMS of MSA that are not included in the NMSS scale) of MSA, were also recorded.

### Statistical analysis

All statistical analyses were performed using SPSS Statistics 25.0 for Windows (SPSS, Chicago, IL, USA). The continuous data were expressed as mean ± standard deviation, whereas the discontinuous data were presented as median values (quartile). Categorical data were expressed as numbers and percentages. Differences in continuous variables between groups were analyzed using the Student's *t*-test or Kruskal-Wallis test, as appropriate. Differences in categorical data between groups were analyzed using SPSS Chi-square or Fisher's exact test. The Spearman's rank correlation coefficient was used to assess the associations between demographics (age, disease duration, BMI, and education) and clinical variables (disease severity assessed by UMSARS-IV for MSA and H&Y stage for PD and PSP; motor symptom severity assessed by UMSARS-II for MSA and UPDRS-III for PD and PSP; and NMS assessed by NMSS). The NMS variables found to be statistically significant in the comparative analysis of MSA vs. PD and PSP were then included in a multinomial logistic regression model with age, sex, disease duration, and education as covariates. Values of *p* < 0.05 were considered statistically significant (two-tailed test).

## Results

### Demographic and clinical characteristics

A total of 178 patients were enrolled in the study, constituting 61 patients in MSA (18 MSA-P and 43 MSA-C), 87 patients in PD, and 30 patients in the PSP group. The demographic and clinical characteristics of the three groups are presented in Table 1. The enrolment age and onset age were significantly younger in MSA than those in PD and PSP (all *p* < 0.05). Sex, education, weight, and BMI did not differ significantly among the three groups, but the height of patients with MSA was significantly higher than PD (*p* < 0.05). Among patients with PD and patients with PSP, the disease severity (assessed by H&Y stage) was poor in PSP than in PD (*p* < 0.05), while motor symptoms (assessed by MDS-UPDRS-III) showed no statistical difference between the two groups. Patients with MSA had preserved cognitive function (higher MMSE scores) than patients with PSP (*p* < 0.05). Patients with MSA were more depressed or full of anxiety (higher HAMD and HAMA scores) compared to patients with PD and PSP (all *p* < 0.05), but there was no difference between patients with PD and PSP. No significant differences were observed in sleep quality (assessed by PSQI scores) among the three parkinsonian patient groups.

**Table 1:** Demographic and clinical data of MSA vs. PD and PSP.

	**MSA (*n* = 61)**	**PD (*n* = 87)**	**PSP (*n* = 30)**	***p1***	**PD + PSP (*n* = 117)**	***p2***
Female/male	20/41	39/48	13/17	0.319	52/65	0.133
Age	56.39 ± 6.92	69.22 ± 9.93	66.33 ± 5.13	<0.001^†^^,^^a^^,^^b^	68.48 ± 9.01	<0.001
Age of onset	52.50 ± 6.70	63.31 ± 10.91	62.86 ± 5.32	<0.001^†^^,^^a^^,^^b^	63.20 ± 9.77	<0.001^†^
Education	11.82 ± 3.25	11.37 ± 4.00	10.43 ± 4.36	0.270	11.13 ± 4.10	0.255
Disease duration	3.81 ± 1.72	6.03 ± 4.44	3.49 ± 2.43	0.001^a^^,^^c^	5.38 ± 4.17	0.004^†^
Height	168.69 ± 7.24	164.94 ± 7.81	166.00 ± 8.66	0.016^a^	165.14 ± 7.94	0.005
Weight	69.16 ± 10.34	66.58 ± 11.77	65.63 ± 10.06	0.285	66.41 ± 11.43	0.122
BMI	24.26 ± 3.03	24.38 ± 3.45	23.71 ± 2.16	0.696	24.26 ± 3.25	0.570^†^
Disease severity^#1^	4.00 (2.00, 4.00)	2.00 (1.50, 3.00)	2.50 (2.00. 3.25)	0.005^c^	2.50 (2.00, 3.00)	NA
Motor symptom^#2^	21.70 ± 8.25	32.08 ± 19.35	32.07 ± 18.07	0.998	32.08 ± 18.96	NA
MMSE	27.48 ± 2.71	25.91 ± 5.92	23.97 ± 4.40	0.039^b^	25.41 ± 5.62	0.080^†^
HAMD	22.07 ± 10.64	7.83 ± 8.29	7.20 ± 7.48	<0.001^†^^,^^a^^,^^b^	7.67 ± 8.07	<0.001^†^
HAMA	16.30 ± 8.62	8.11 ± 8.42	7.47 ± 6.95	<0.001^a^^,^^b^	7.95 ± 8.04	<0.001
PSQI	8.84 ± 4.54	7.44 ± 4.67	7.60 ± 5.82	0.203	7.47 ± 4.87	0.075

*p1* represents *p*-value of the comparison among three groups of MSA, PD, and PSP; *p2* represents *p*-value of the comparison of MSA group vs. PD+PSP group. Gender differences among groups were compared using SPSS-chi-square test. Unless otherwise specified, comparisons of continuous variables between three groups were performed using SPSS-analysis of variance, and comparisons of continuous variables between two groups were performed using SPSS-Student's t-test.

† Kruskal-Wallis test was used to detect group differences.

a Significant differences between MSA and PD;

b Significant differences between MSA and PSP;

c Significant differences between PD and PSP. NA, not applicable due to different assessment scales used.

MSA, multiple system atrophy; PD, Parkinson's disease; PSP, progressive supranuclear palsy; UMSARS-IV, the unified multiple system atrophy rating scale part IV; UMSARS-II, the unified multiple system atrophy rating scale part II; UPDRS-III, movement disorders society–revised unified Parkinson's disease rating scale part III; H&Y stage, Hoehn and Yahr stage; MMSE, mini-mental state examination; HAMD, hamilton rating scale for depression; HAMA, hamilton anxiety scale; PSQI, Pittsburgh sleep quality index; NMSS, the non-motor symptoms scale.

#1 Disease severity was assessed by H&Y stage for PD and PSP, UMSARS-IV for MSA;

#2 Motor symptom was assessed by UPDRS-III (on)for PD and PSP, UMSARS-II for MSA.

### Frequency of NMS in MSA, PD, and PSP

**Figure 1:**
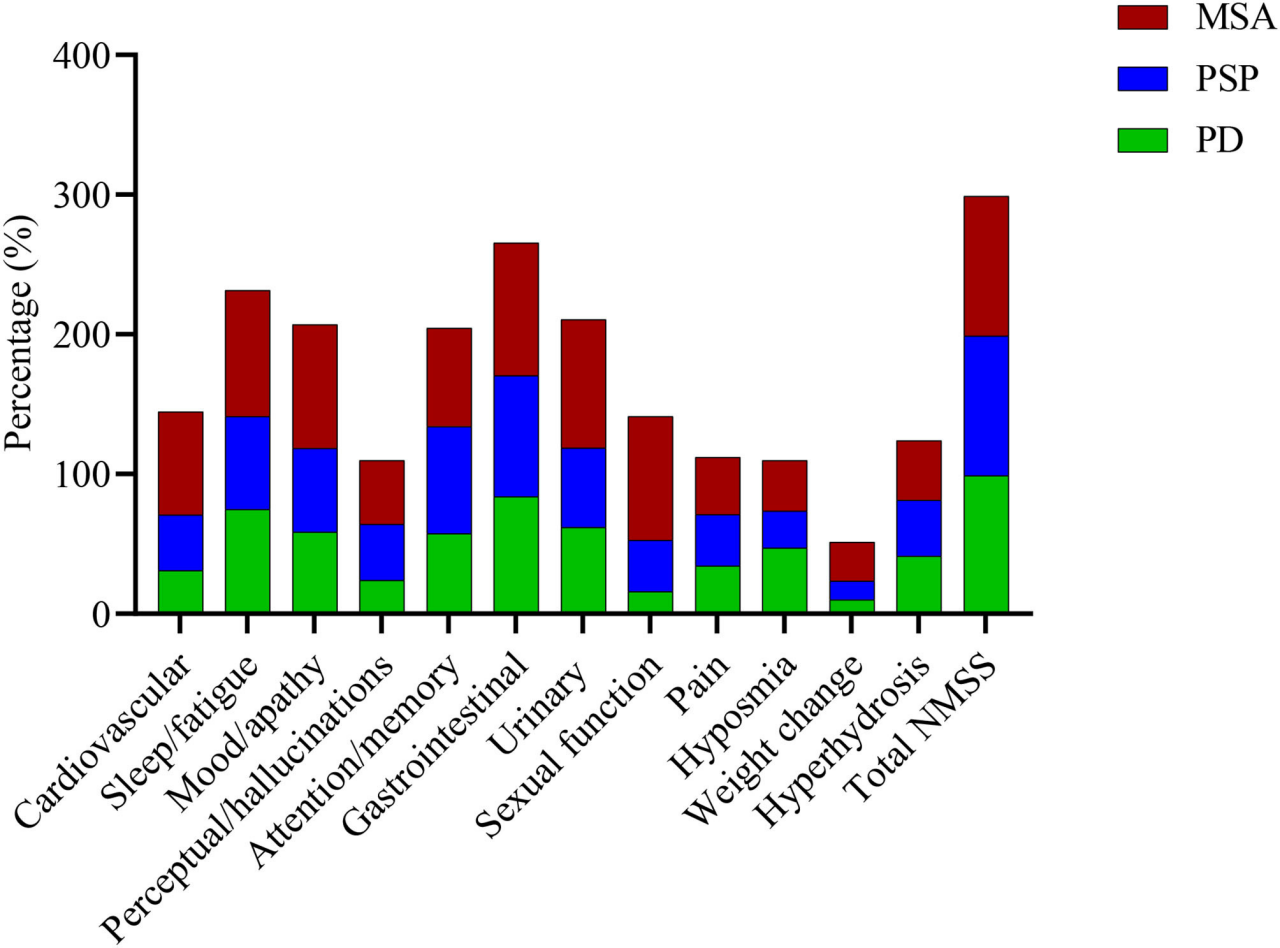
Frequencies of non-motor symptoms in the MSA, PD, and PSP groups. There were about similar frequencies of total NMSS, attention/memory, and gastrointestinal subdomains symptoms among the three parkinsonian disorders. However, symptoms of the cardiovascular, sleep/fatigue, mood/apathy, urinary, and sexual function subdomains and weight change were more commonly seen in patients with MSA than PD and PSP. MSA, multiple system atrophy; PD, Parkinson's disease; PSP, progressive supranuclear palsy; NMSS, the non-motor symptom scale.

About 100% of patients with MSA had at least one non-motor symptom. The most frequently affected subdomains of NMS in MSA were gastrointestinal (95.1%), urinary (91.8%), sleep/fatigue (90.2%), sexual function (88.5%), and mood/apathy (88.5%) (Supplementary Table 1, Figure 1). Based on mean NMSS subdomain scores, the most affected subdomains of NMSS were mood/apathy, urinary, sleep/fatigue, sexual function, and gastrointestinal (Supplementary Table 1). The most prevalent individual NMS were constipation (83.6%), followed by problems having sex (82.0%), swallowing (82.0%), lack of motivation (82.0%), and loss of sexual interest (80.3%). In the PD group, 98.9% of patients had at least one non-motor symptom. The most prevalent subdomains of NMSS were gastrointestinal (83.9%), miscellaneous (77.0%), sleep/fatigue (74.4%), urinary (62.1%), and mood/apathy (58.6%). Based on mean NMSS subdomain scores, the most affected subdomains of NMSS in PD were miscellaneous, sleep/fatigue, gastrointestinal, urinary, and mood/apathy. The most prevalent individual NMS were constipation (70.1%), followed by forgetting things or events (55.2%), depression (48.3%), nocturia (47.1%), and hyposmia (47.1%). In the PSP group, all patients suffered from at least one non-motor symptom. The most prevalent subdomains of NMSS were gastrointestinal (86.7%), miscellaneous (76.7%), attention/memory (76.7%), sleep/fatigue (66.7%), and mood/apathy (60.0%). According to mean NMSS subdomain scores, the most affected subdomains of NMSS in PSP were mood/apathy, sleep/fatigue, gastrointestinal, attention/memory, and urinary. The most prevalent individual NMS were forgetting things or events (73.3%), followed by constipation (60.0%), swallowing (56.7%), fatigue (50.0%), and concentration (46.7%).

### Associations of NMS with other clinical characteristics

**Figure 2:**
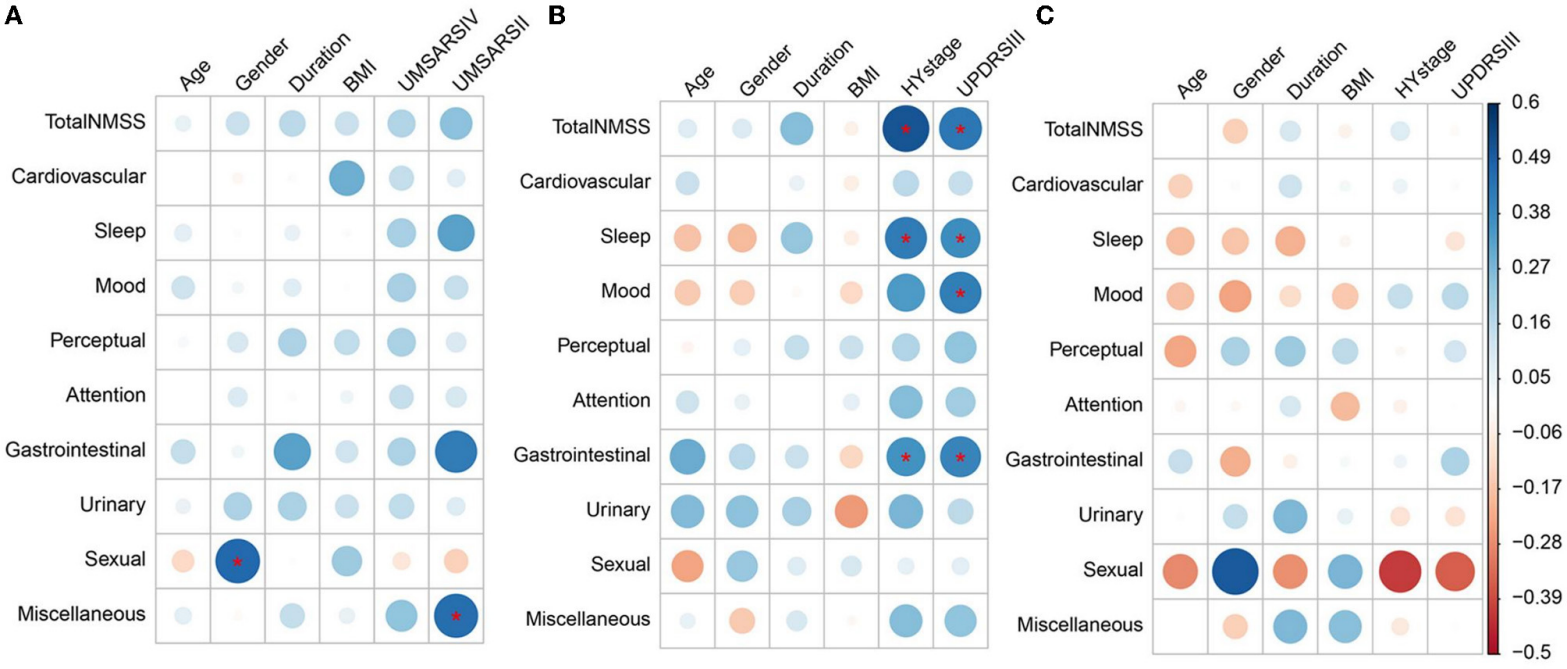
Heatmap of non-motor symptoms correlation with other clinical features in MSA, PD, and PSP. **(A–C)** Correspond to the left, middle, and right parts of the graph, respectively, and represent the correlations of non-motor symptoms with clinical features in MSA, PD, and PSP, respectively. **(A)** Sexual dysfunction subdomain scores in MSA were positively correlated with the male gender. Miscellaneous subdomain scores of MSA were positively correlated with UMSAR-II scores in MSA. **(B)** Total NMSS scores, sleep/fatigue, and gastrointestinal subdomain scores of PD were positively correlated with H&Y stage. Total NMSS scores, sleep/fatigue, mood/apathy, and gastrointestinal subdomain scores of PD were positively correlated with UPDRS-III. **(C)** No significant correlations were found between the total and all subdomain scores of the NMSS of PSP with age, gender, disease duration, BMI, H&Y stages, and UPDRS-III scores. Blue and red colors in each figure represented positive or negative correlations between non-motor symptoms and the clinical features, respectively. The colors in the ribbon on the right indicate the magnitude of the correlations, the same color represents the same correlation coefficient in the whole graph. *Statistically significant correlations after Bonferroni's correction (10 NMS parameters, 6 clinical features parameters; adjusted significance level at *p* < 0.00083). MSA, multiple system atrophy; PD, Parkinson's disease; PSP, progressive supranuclear palsy; UMSARS-IV, the unified multiple system atrophy rating scale, Part IV; UMSARS-II, the unified multiple system atrophy rating scale, Part II; H&Y stage, Hoehn and Yahr stage; UPDRS-III, movement disorders society–revised unified Parkinson's disease rating scale, Part III; NMSS, the non-motor symptom scale; NMS, non-motor symptoms.

As illustrated in Figure 2 and Supplementary Table 2, after Bonferroni's corrections, Spearman's correlations showed that the sexual function subdomain score was positively correlated with male gender (*r* = 0.466), the miscellaneous subdomain score was positively correlated with the UMSAR-II score (*r* = 0.456), and the total and all subdomain scores of NMSS were independent of UMSAR-IV scores in MSA group. In addition, the total NMSS score (*r* = 0.515), sleep/fatigue (*r* = 0.414), and gastrointestinal (*r* = 0.354) subdomain scores were positively correlated with H&Y stage, and the total NMSS score (*r* = 0.430), sleep/fatigue (*r* = 0.371), mood/apathy (*r* = 0.410), and gastrointestinal (*r* = 0.392) subdomain scores were positively correlated with UPDRS-III in the PD group. No significant correlations were found between the total and all subdomain scores of the NMSS of PSP with demographics, disease severity, and motor symptoms after Bonferroni's corrections.

### Comparison of NMS in MSA with those in PD and PSP

**Figure 3:**
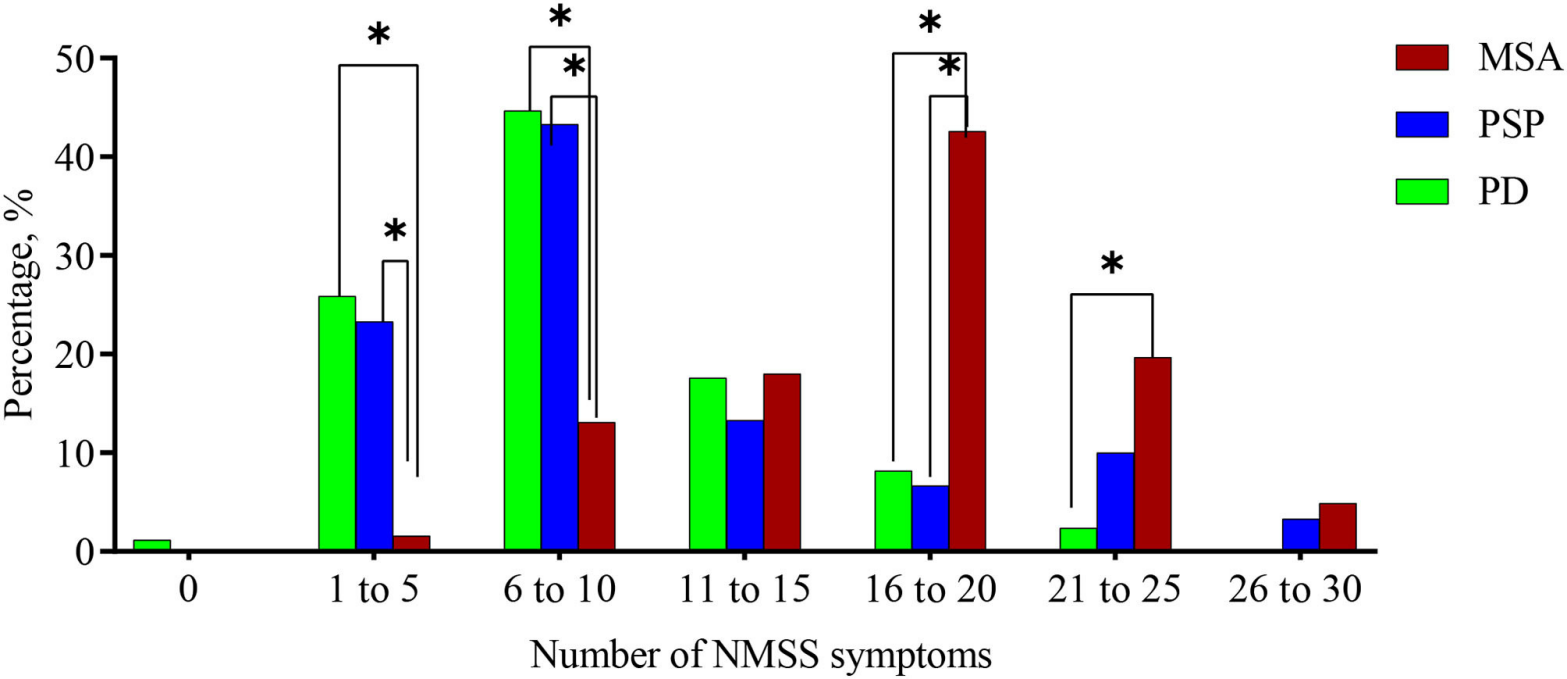
Distribution of the number of non-motor symptoms in MSA, PD, and PSP. The proportion of patients with 16–20 non-motor symptoms was higher in the MSA group than in PD and PSP groups (all *p* < 0.05); There was a higher proportion of patients with 21–25 non-motor symptoms in MSA than in PD (*p* < 0.05). The proportion of patients with 1–5 or 6–10 non-motor symptoms was higher in the PD and PSP groups compared to the MSA group (all *p* < 0.05). ^*^Represents *p*-value ≤ 0.05 between two disease entities. MSA, multiple system atrophy; PD, Parkinson's disease; PSP, progressive supranuclear palsy.

The mean of total NMSS scores and the mean number of NMS for MSA, PD, and PSP were 82.15 ± 46.10, 36.14 ± 30.78, 50.30 ± 55.05, 16.90 ± 5.59, 8.66 ± 4.43, and 11.03 ± 7.16, respectively. The MSA group had higher total NMSS scores (Supplementary Table 1) and NMS numbers than PD and PSP (*p* < 0.001 for all). Regarding the number distribution of NMS, 85.2% of patients with MSA had more than 10 NMS, which was significantly higher than that of PD (28.7%) and PSP (33.3%) (Figure 3, Supplementary Table 3, *p* < 0.001 for all). In NMSS subdomains, the frequency and scores of cardiovascular, sleep/fatigue, mood/apathy, urinary, and sexual function subdomains were significantly higher in MSA than in PD or PSP (All *p* < 0.05) (Supplementary Table 1, Figure 1). The frequencies and scores of individual NMS among MSA, PD, and PSP are shown in Supplementary Table 1.

### Multinominal logistic regression analysis comparing NMS between MSA, PD, and PSP

The individual NMS showing significant group differences as described above were then entered into a multinomial logistic regression, with age, gender, disease duration, and education as covariates. Patients with fainting (OR = 40.35, 95%CI 3.81–427.75; *p* = 0.002), lack of motivation (OR = 13.71, 95%CI 2.60–72.40; *p* = 0.002), swallowing (OR = 8.50, 95%CI 1.35–53.50; *p* = 0.023), and loss of sexual interest (OR = 8.56, 95%CI 1.35–54.28; *p* = 0.023) were more likely to be associated with MSA compared to PD (Table 2). Patients with a lack of motivation (OR = 25.81, 95%CI 3.14–211.88; *p* = 0.038) were more likely to be associated with MSA, while patients with forgetting things or events (OR = 0.07, 95%CI 0.011–0.503; *p* = 0.008) were less likely to be associated with MSA compared to PSP (Table 2). When considering PD and PSP as one group, patients experiencing fainting (OR = 6.87, 95%CI 1.51–31.23; *p* = 0.013), lack of motivation (OR = 87.76, 95%CI 9.44–743.37; *p* < 0.001), swallowing (OR = 8.09, 95%CI 1.69–38.86; *p* = 0.009), and loss of sexual interest (OR = 4.57, 95%CI 1.01–20.65; *p* = 0.049) were more likely to be associated with MSA, and patients with loss concentration (OR = 0.13, 95%CI 0.02–0.88; *p* = 0.037) and forgetting things or events (OR = 0.05, 95%CI 0.01–0.37; *p* = 0.003) were less likely to be associated with MSA (Table 2).

**Table 2:** Multinominal logistic regression analysis of non-motor symptoms between MSA, PD, and PSP.

**Variables**	**Multivariate analysis**		
	β	**aOR (95CI%)**	***p***
**MSA vs. PD (Ref)**			
Falls because of fainting	3.698	40.353 (3.807–427.749)	0.002
Lack motivation	2.618	13.714 (2.598–72.402)	0.002
Swallowing	2.140	8.495 (1.349–53.499)	0.023
Loss of sexual interest	2.147	8.556 (1.349–54.281)	0.023
**MSA vs. PSP (Ref)**			
Lack motivation	3.251	25.805 (3.143–211.879)	0.038
Forget things or events	−2.618	0.073 (0.011–0.503)	0.008
**MSA vs. PD** + **PSP (Ref)**			
Falls because of fainting	1.927	6.871 (1.512–31.225)	0.013
Lack motivation	4.428	83.757 (9.437–743.368)	<0.001
Swallowing	2.091	8.092 (1.685–38.856)	0.009
Loss of sexual interest	1.519	4.566 (1.010–20.647)	0.049
Concentration	−2.065	0.127 (0.018–0.883)	0.037
Forget things or events	−2.933	0.053 (0.008–0.367)	0.003

Multiple logistic regression adjusted for age, gender, disease duration and education.

MSA, multiple system atrophy; PD, Parkinson's disease; PSP, progressive supranuclear palsy.

### The onset of NMS relative to the onset of motor symptoms in MSA

**Figure 4:**
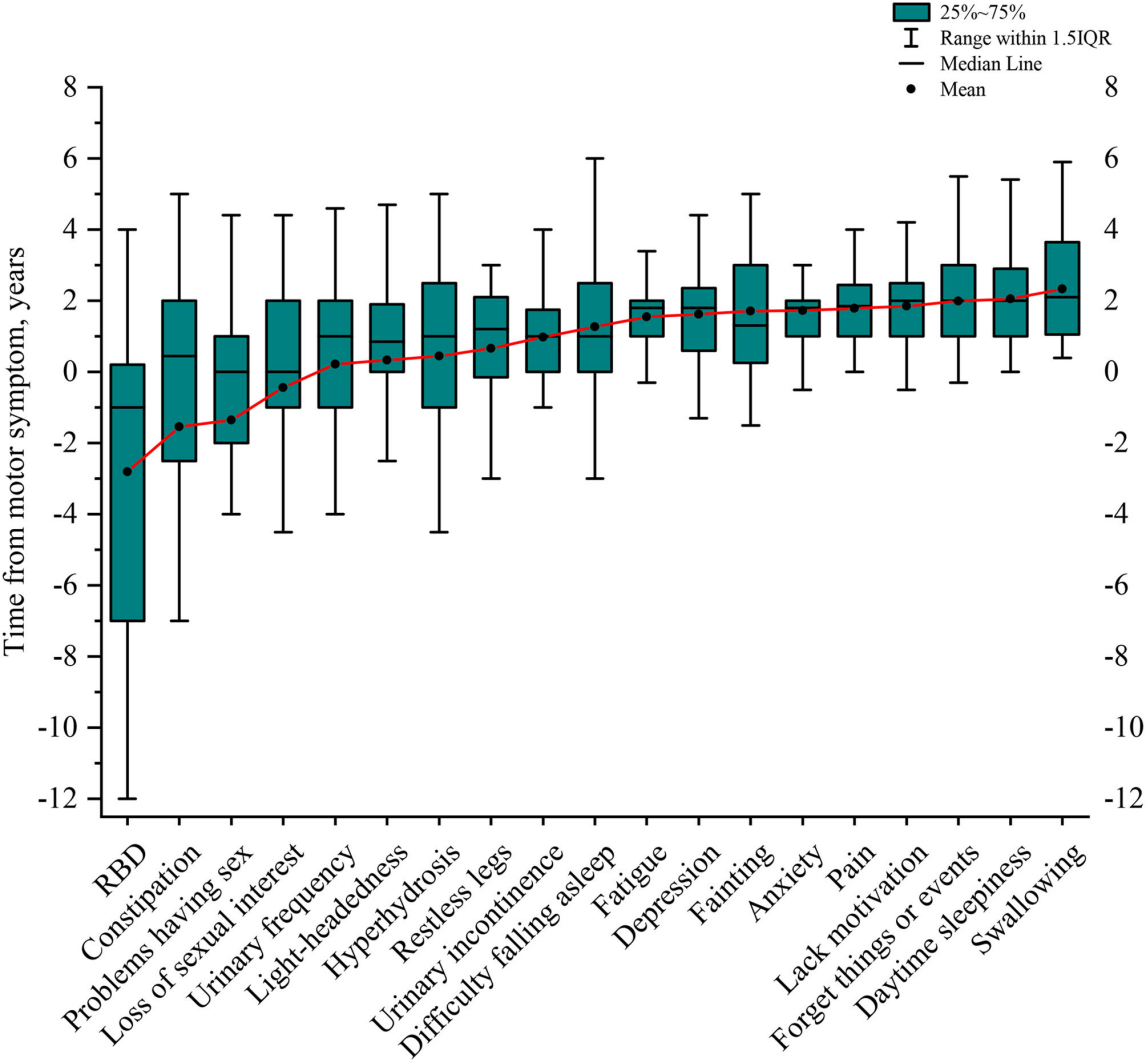
Timeline of NMS presence in relation to the onset of motor symptoms in MSA. RBD was the earliest sign, followed by constipation, sexual problems, and a loss of sexual interest before motor symptoms started. The following NMS started nearly at the same time as motor symptoms, including urinary frequency, light-headedness, hyperhidrosis, restless legs, and urinary incontinence. As the disease progresses, patients with MSA might have difficulty falling asleep, fatigue, depression, fainting, anxiety, pain, lack of motivation, forgetting things or events, daytime sleeping, and develop swallowing difficulties at the late stage of the disease. NMS, non-motor symptoms; MSA, multiple system atrophy; RBD, REM sleep behavior disorder.

After identifying the specific NMS for MSA through multinominal logistic regression, we investigated the presentation timing of those NMS in relation to the onset of motor symptoms. All patients with MSA included in the study presented with motor symptoms, and 45 (73.8%) patients initially presented with gait or postural instability, 15 (24.6%) initially with bradykinesia, and 1 (1.6%) initially with resting tremor. The spans of NMS in MSA relative to the onset of motor symptoms in patients with MSA were listed in the order of their chronological occurrence (Figure 4, Supplementary Table 4). RBD signs, constipation, problems having sex, and loss of sexual interest preceded the onset of motor symptoms by a mean of 2.81 ± 4.51, 1.54 ± 6.32, 1.35 ± 4.70, and 0.45 ± 3.61 years, respectively (Figure 4, Supplementary Table 4). In the urinary domain, the mean onset time of urinary frequency, urinary urgency, nocturia, and urinary incontinence was 0.22 ± 2.97, 0.24 ± 2.74, 0.67 ± 2.64, and 0.98 ± 1.45 years, respectively, after the onset of motor symptoms (Supplementary Table 4). In the cardiovascular domain, light-headedness and falls because of fainting occurred after the onset of motor symptoms at a mean of 0.33 ± 3.78 and 1.71 ± 1.64 years, respectively (Figure 4, Supplementary Table 4). With the exception of concentration, hyperhidrosis, and restless legs syndrome, most other NMS occurs 1–2 years after the onset of motor symptoms (Supplementary Table 4).

## Discussion

To the best of our knowledge, this is the first study that systematically compares the frequency and severity of NMS among MSA, PD, and PSP, and the first study that systematically investigates the timing of NMS presentation in MSA. This study found that the spectrum of NMS in MSA was different from those in PD and PSP. Furthermore, patients with MSA had a significantly higher burden of NMS than patients with PD and PSP. Finally, this study found RBD, constipation, problems having sex, and loss of sexual interest preceded the onset of motor symptoms in MSA and may be clinical markers for prodromal MSA.

This study demonstrated that almost all patients with MSA, PD, and PSP experienced at least one non-motor symptom, which was consistent with previous studies (17, 32). Based on mean NMSS subdomain scores, this study found that the most affected NMS subdomains in MSA were in the order of mood/apathy, urinary, sleep/fatigue, sexual function, and gastrointestinal. Similar to a previous study, which was based on mean NMSS subdomain scores, the most affected NMSS subdomains in MSA was in the order of urinary, mood/apathy, sleep/fatigue, sexual function, and gastrointestinal (17). In addition, based on the mean NMSS subdomain scores, this study found that the most affected NMSS subdomains in PD were in the order of miscellaneous, sleep/fatigue, gastrointestinal, urinary, mood/apathy, and the most affected NMSS subdomains in PSP were in the order of mood/apathy, sleep/fatigue, gastrointestinal, attention/memory, and urinary. However, Lee et al. (32) found that the most affected NMSS subdomains for PD were in the order of mood/apathy, attention/memory, sleep/fatigue, urinary, and sexual function, and for PSP were in the order of attention/memory, mood/apathy, sleep/fatigue, urinary, and cardiovascular. The discrepancy may lie in disease duration, race, and sample size between this study and Lee et al.'s (32) study.

We found that there were gender differences in sexual function subdomain scores, and the sexual function subdomain symptoms were more prominent in male patients with MSA. Previous studies have shown that erectile dysfunction is common among male patients with MSA (43, 52). Female sexual dysfunction has been less studied in α-synucleinopathies; however, a previous study systematically assessed sexual dysfunction in female patients with MSA and found that the prevalence of this disorder was higher among them than in age-matched controls (53). The results of this study suggest that sexual dysfunction is more common in male patients with MSA, which may be attributed to different clinical attention given to the male or female sexual function. Evaluators often paid more attention to sexual dysfunction symptoms in male than in female, leading to underestimating sexual dysfunction in female.

Consistent with previous studies (54, 55), we found that the total NMSS scores were positively correlated with UPDRS-III scores and H&Y stages in PD, suggesting that NMS worsened with the motor symptoms aggravation and the disease progression. After Bonferroni's correction, the sleep/fatigue subdomain and gastrointestinal domain scores were positively correlated with H&Y stages in PD, indicating that sleep/fatigue and gastrointestinal domain symptoms of PD deteriorated with the disease progression. The sleep/fatigue, mood/apathy, and gastrointestinal subdomain scores were positively correlated with UPDRS-III scores in PD after Bonferroni's correction, while the perceptual/hallucinations and miscellaneous domain scores were positively correlated with UPDRS-III scores before Bonferroni's correction, which was generally consistent with a previous study (56). In MSA, we found that the total and all subdomain scores of NMSS in MSA were independent of UMSAR-IV scores, suggesting that the NMS of MSA was not associated with disease severity. In addition, total NMSS scores were independent of UMSAR-II scores, and actually, the majority of NMSS subdomains of MSA were not correlated with UMSAR-II scores, except that the miscellaneous subdomain was associated with motor symptoms. Different from PD, the total NMSS score and the majority of NMSS subdomains score of MSA were not correlated with disease severity and motor symptoms, which may be attributed to different constitutional weights of NMS in MSA and PD. On the contrary, the current UMSARS scale used in this study may not precisely measure motor symptoms and disease severity in patients with MSA, as a ceiling effect and a lack of correlation with disease severity have been implicated in UMSARS (57–59). In PSP, after Bonferroni's correction, the total and all subdomain scores of NMSS were independent of UPDRS-III scores and H&Y stages. Our findings were consistent with the studies by Chaithra et al. (18), and Ou et al. (20), which showed that total and all subdomain scores of PSP were not associated with UDPDS-III scores. The results of this study indicate that NMS of PSP was not associated with either the disease or the motor symptoms severities.

This study showed there were higher total NMSS scores in MSA than in PD or PSP, indicating that patients with MSA had a higher burden of NMS than patients with PD or PSP. However, the study by Lee et al. (32) showed that the burden of NMS in MSA was similar to that in PD and PSP. The difference between the two studies might be attributed to different proportions of MSA-P and MSA-C in the study cohorts. In this study, patients with MSA-C accounted for 70.0% of patients with MSA, whereas in Chan-Nyoung Lee's study, patients with MSA-C accounted only for 30.0% of patients with MSA. This study found that patients with MSA had a higher number of NMS than patients with PD and PSP, which was consistent with a previous study (60). Comparing the number distribution of NMS in MSA, PD, and PSP, we found that the number of NMS in patients with MSA was mostly distributed in more than 10, while the numbers of NMS in patients with PD and PSP were mostly distributed in 10 or below. To the best of our knowledge, this is the first study demonstrating the NMS number distribution pattern in MSA, PD, and PSP. In NMSS subdomains, the frequencies and NMSS subdomain scores of cardiovascular, sleep/fatigue, mood/apathy, urinary, and sexual function were significantly higher in MSA than in PD and PSP. A study by Colosimo et al. (60) used a non-motor symptom cluster questionnaire to assess NMS and found that patients with MSA had a higher incidence of urinary symptoms and orthostatic symptoms than patients with PD and PSP, and patients with MSA had a higher incidence of fatigue and apathy than patients with PD, which were consistent with our findings. However, Colosimo et al.'s (60) study also showed that the frequency of sleep disturbances in MSA was similar to those in PD and PSP, while sleep problems were more often seen in patients with MSA in this study. Combining the differences in the number distribution of NMS among MSA, PD, and PSP, and the differences in the burden of multiple NMSS subdomains among MSA, PD, and PSP, it could be concluded that the spectrum of NMS in MSA was different from those in PD and PSP.

There were higher frequencies and scores for many individual NMS in MSA than those in PD and PSP, except for forgetting things or events. Multivariable analysis revealed that, compared with PD, those who had fainting, a lack of motivation, swallowing, and a loss of sexual interest were more likely to be diagnosed with MSA. Fainting is a severe symptom caused by orthostatic hypotension (61). Previous studies had shown that patients with MSA had a higher incidence of orthostatic hypotension than patients with PD (62, 63), which was consistent with the findings in this study. Magalhaes et al. (63) retrospectively analyzed autonomic dysfunction in pathologically confirmed PD and MSA and found that MSA had a higher incidence of dysphagia than PD, which supported our findings. Endoscopic evaluation of swallowing in patients with MSA and PD by Vogel et al. (64) found that patients with MSA exhibited more symptoms suggestive of oral-phase disturbances (premature spillage, swallowing debris) than patients with PD, which could be the main phenotype of dysphagia in MSA. We found that the incidence of sexual dysfunction was higher in MSA than in PD, which was partially in agreement with Garg et al. (65), who used SCOPA-AUT to assess autonomic dysfunction in MSA and PD and found the incidence of sexual dysfunction in MSA was higher than in PD in males, although this difference did not reach statistical significance. Patients with MSA were more likely to suffer from a lack of motivation, while patients with PSP were more likely to suffer from forgetting things or events. While previous studies have shown that postural hypotension is more common in patients with MSA than in patients with PSP, which helps to distinguish MSA from PSP (30, 31). Compared with PD and PSP patients, patients with MSA were more likely to suffer from falls because of fainting, lack of motivation, swallowing, and loss of sexual interest, but less likely to suffer from loss of concentration and forgetting things or events. The study by Santangelo et al. (22) showed that immediate recall and executive function impairment in PSP were more obvious than those in MSA and PD, and immediate recall and executive dysfunction in MSA and PD were similar, which could explain why the PSP group or PD and PSP group in this study were more likely to have forgotten things or events than the MSA group. Santangelo et al. (22) found that patients with PSP had poorer performance in the Stroop test than patients with MSA and PD, and patients with PD had similar Stroop test performance to patients with MSA, which indicated that patients with PSP had poor attention than patients with PD and MSA. Similarly in this study, we showed that PD and PSP groups were more likely to have inattention than the MSA group. By systematically assessing the timing of NMS presentation relative to the onset of motor symptoms in patients with MSA, we found that RBD, constipation, problems having sex, and loss of sexual interest preceded the onset of motor symptoms with a maximum of 7 years. While urinary symptoms and orthostatic symptoms occurred within 1 year after the onset of motor symptoms, most of the remaining NMS occurred within 2 years after the onset of motor symptoms. In this study, RBD was the first symptom of MSA, which precede motor symptoms onset by a mean of 2.81 ± 4.51 years (median, 1 year). Previous studies have indicated that RBD is likely the first symptom of MSA (40, 44), and one study has shown that RBD precedes the disease onset with a median time of 3 (2–5) years (40). These studies were generally consistent with our findings. A previous clinicopathological study found symptomatic orthostatic hypotension occurred within the first year after disease onset in MSA (31), which also supports our findings. Our findings are also in accordance with the diagnostic criteria for MSA (2022 version), in which RBD, orthostatic hypotension, and urogenital failure were clinical indicators of possible prodromal MSA (47). This study expanded on the work of McKay and Cheshire (43), who investigated the timing of the onset of NMS relative to diagnosis in 30 patients with MSA. However, they did not systematically assess NMS using the NMSS scale and did not investigate the timing of NMS presentation relative to motor symptoms onset.

### Limitations and future directions

First, the sample sizes of MSA and PSP in this study were small. There were only 18 patients with MSA-P, which was insufficient for subgroup analysis. Future studies were required to explore whether the burden of NMS differs between MSA-P and MSA-C and confirm the NMS appearance order in larger MSA cohorts. Second, NMS was investigated only by means of a questionnaire; objective methods, including an olfactory test; and autonomic testing were not used in this study. Third, autonomic symptoms are an important feature of Parkinsonian disorders and impact QoL. These autonomic symptoms increase with age, disease severity, and medication use. Their severities were also associated with more motor dysfunction, depressive symptoms, cognitive dysfunction, psychiatric complications, and sleep disturbances. For a better evaluation of these aspects, a specific questionnaire investigating only autonomic dysfunctions (SCOPA-AUT or COMPASS-31) should be used. Fourth, RBD was diagnosed by questionnaires. However, a questionnaire could misdiagnose an RBD diagnosis as other sleep disorders could mimic RBD. The certainty of an RBD diagnosis could be performed only by means of video polysomnography. On the contrary, some studies have reported RBD diagnosis as a video-polysomnographic finding in patients with MSA with a negative symptomatic history of this sleep disorder. Finally, the timing and latency of NMS onset in the MSA group were retrospectively, but not prospectively, collected, therefore recall bias could impact results.

## Conclusion

We found the spectrum of NMS in MSA is different from those in PD and PSP. Patients with MSA have a significantly higher NMS burden than patients with PD or PSP. RBD, constipation, problems having sex, and loss of sexual interest precede the onset of motor symptoms in MSA, which could be clinical indicators for an early diagnosis of MSA.

## Data availability statement

The original contributions presented in the study are included in the article/Supplementary material, further inquiries can be directed to the corresponding author.

## Ethics statement

The studies involving human participants were reviewed and approved by the Institutional Review Board/Ethics Committee of Beijing Tiantan Hospital, Capital Medical University, Beijing. The patients/participants provided their written informed consent to participate in this study.

## Author contributions

WZH drafted and revised the manuscript, conducted clinical assessments with patients, and did data collection and data analysis. LXC and JHY conducted clinical assessments on patients and collected data. XSZ, YSP, WHG, JHM, and ZRW were involved in participants' recruitment, clinical diagnoses, and clinical definitions of different symptoms. YH conceived and designed the manuscript, performed patient recruitment and critical revision, and approved the final manuscript. All authors contributed to the article and approved the submitted version.
