# Supplementary material for: Non-motor symptoms in multiple system atrophy: A comparative study with Parkinson's disease and progressive supranuclear palsy
Source: Front Neurol. 2023 Jan 23;13:1081219. doi: 10.3389/fneur.2022.1081219 (PMC9901543; doi:10.3389/fneur.2022.1081219)
Supplement: Supplementary file 1 [file Data_Sheet_1.docx]

Supplementary Material

**Patient Enrollment:**

190 patients with MSA patients were registered with Beijing Tiantan Brain Health Group - Patient Education Initiative, 105 of them provided clinical records during their past visits to different hospitals, mainly from China-Japan friendship hospital. Among these 105 patients, 69 patients were clinically assessed by our team members, and 56 patients who had complete clinical dataset and consensus of clinical diagnosis of MSA were included in this study. Five other MSA patients were referred from Aerospace Center Hospital. PD patients were recruited from two hospitals, including 47 patients from the neurology outpatient department of Beijing Tiantan Hospital and 40 referrals from Aerospace Center Hospital. PSP patients were recruited from three hospitals, including 15 patients from the neurology outpatient department of Beijing Tiantan Hospital, 5 referrals from Aerospace Center Hospital and 10 referrals from Xuanwu Hospital of Capital Medical University in Beijing. All patients were assessed by a neurologist specializing in movement disorders, and their clinical diagnosis were confirmed by at least one other neurologist from Beijing Tiantan Hospital.

**Comparison of the frequency and score of individual NMS among MSA, PD, and PSP:**

In individual NMS, the frequency and scores of orthostatic symptoms, falls because of fainting, fatigue, restless legs, lost interest in surroundings, lack motivation, depression, urinary urgency, urinary frequency, nocturia, loss of sexual interest, problems having sex were significantly higher in MSA than in PD or PSP (both *p*<0.05) (Supplementary Table 1). In addition, the frequencies and scores of flat mood, difficulty in experiencing pleasure, double vision, loss concentration or swallowing ability were significantly higher in MSA than in PD or PSP (both *p*<0.05) (Supplementary Table 1). Compared with PSP, MSA had lower frequency and score of forget things or events and higher frequency and score of constipation (*p*<0.05 for all) (Supplementary Table 1).

**Supplementary Table 1 | Frequency of NMS and NMSS scores in patients with MSA vs PD and PSP**

| **NMSS items** | **Frequency of NMS, n, (%)** | | | | ***p*1** | ***p*c1** | **NMSS scores** | | | | ***p*2** | ***P*c2** |
| --- | --- | --- | --- | --- | --- | --- | --- | --- | --- | --- | --- | --- |
|  | **MSA (n=61)** | **PD (n=87)** | **PSP (n=30)** | **PD+PSP (n=117)** |  |  | **MSA (n=61)** | **PD (n=87)** | **PSP (n=30)** | **PD+PSP (n=117)** |  |  |
| **Total NMSS** | **61(100)** | **86(98.9)** | **30(100)** | **116(99.1)** | **0.487** | **1.000** | **82.15±46.10** | **36.14±30.78** | **50.30±55.05** | **39.77±38.71** | **<0.001^†,a,b^** | **<0.001** |
| **Cardiovascular** | **45(73.8)** | **27(31.0)** | **12(40)** | **39(33.3)** | **<0.001^a,b^** | **<0.001** | **4.56±4.34** | **1.13±2.80** | **4.03±7.20** | **1.87±4.52** | **<0.001^†,a,b^** | **<0.001** |
| Ort.sym. | 42(68.9) | 25(28.7) | 12(40) | 37(31.6) | <0.001^a,b^ | <0.001 | 3.54±3.72 | 0.87±2.11 | 2.47±4.06 | 1.28±2.81 | <0.001^†,a,b^ | <0.001 |
| F.B.F. | 32(52.5) | 6(6.9) | 6(20) | 12(10.3) | <0.001^a,b^ | <0.001 | 1.02±1.43 | 0.25±1.21 | 1.57±3.54 | 0.59±1.13 | <0.001^†,a,b^ | 0162 |
| Sleep/Fatigue | 55(90.2) | 65(74.7) | 20(66.7) | 85(72.6) | 0.017^a,b^ | 0.007 | 12.87±10.91 | 6.64±8.18 | 8.00±11.36 | 6.99±9.07 | <0.001^†,a,b^ | <0.001 |
| D.S. | 30(49.2) | 35(40.2) | 11(36.7) | 46(39.3) | 0.425 | 0.207 | 1.85±2.94 | 1.45±2.70 | 1.70±3.11 | 1.51±2.80 | 0.691 | 0.451 |
| Fatigue | 46(75.4) | 36(41.4) | 15(50) | 51(43.6) | <0.001^a,b^ | <0.001 | 5.16±4.58 | 2.00±3.23 | 2.67±4.03 | 2.17±3.45 | <0.001^†,a,b^ | <0.001 |
| Dif.F.Asl. | 39(63.9) | 33(37.9) | 12(40) | 45(38.5) | 0.005^a,b^ | 0.001 | 2.79±3.42 | 2.20±3.55 | 2.70±4.54 | 2.32±3.81 | 0.593 | 0.428 |
| Restless legs | 33(54.1) | 20(23.0) | 7(23.3) | 27(23.1) | <0.001^a,b^ | <0.001 | 3.07±3.86 | 1.00±2.56 | 0.93±1.86 | 0.98±2.39 | <0.001^†,a,b^ | <0.001 |
| **Mood/apathy** | **54(88.5)** | **51(58.6)** | **18(60.0)** | **69(59.0)** | **<0.001^a,b^** | **<0.001** | **18.43±16.93** | **4.51±7.62** | **8.23±12.88** | **5.46±9.34** | **<0.001^†,a,b^** | **<0.001** |
| L. I. S. | 41(67.2) | 20(23.0） | 10(33.3) | 30(25.6) | <0.001^a,b^ | <0.001 | 3.03±3.53 | 0.64±1.64 | 1.27±2.21 | 0.80±1.82 | <0.001^†,a,b^ | <0.001 |
| Lack motivation | 50(82.0) | 11(12.6) | 9(30) | 20(17.1) | <0.001^a,b,c^ | <0.001 | 4.79±4.45 | 0.43±1.42 | 1.27±2.48 | 0.64±1.78 | <0.001^†,a,b^ | <0.001 |
| Anxiety | 36(59.0) | 39(44.8) | 10(33.3) | 49(41.9) | 0.052 | 0.030 | 2.75±3.52 | 1.37±2.35 | 1.37±2.98 | 1.37±2.51 | 0.019^†,b^ | 0.045 |
| depression | 45(73.8) | 42(48.3) | 10(33.3) | 52(44.4) | <0.001^a,b^ | <0.001 | 4.05±4.00 | 1.60±2.46 | 0.90±1.90 | 1.42±2.34 | <0.001^†,a,b^ | <0.001 |
| Flat mood | 33(54.1) | 4(4.6) | 10(33.3) | 14(12.0) | <0.001^a,c^ | <0.001 | 1.57±2.14 | 0.26±1.29 | 1.43±2.99 | 0.56±1.93 | <0.001^†,a,c^ | <0.001 |
| Dif.Ex.Plea., | 38(62.3) | 3(3.4) | 11(36.7) | 14(12.0) | <0.001^a,b,c^ | <0.001 | 2.23±3.37 | 0.21±1.29 | 2.00±3.50 | 0.67±2.22 | <0.001^†,a,c^ | <0.001 |
| **P./H.** | **28(45.9)** | **21(24.1)** | **12(40.0)** | **33(28.2)** | **0.018^a^** | **0.018** | **1.28±2.00** | **0.98±2.74** | **2.77±5.82** | **1.44±3.83** | **0.027^†,a^** | **0.764** |
| Hallucinations | 7(11.5） | 14(16.1) | 6(20) | 20(17.1) | 0.536 | 0.321 | 0.11±0.32 | 0.39±1.49 | 1.17±3.00 | 0.59±2.00 | 0.388^†^ | 0.870 |
| Delusions | 7(11.5) | 4(4.6) | 6(20) | 10(8.5) | 0.044^c^ | 0.528 | 0.20±0.81 | 0.18±1.07 | 0.47±1.50 | 0.26±1.20 | 0.440 | 0.727 |
| Double vision | 22(36.1) | 9(10.3) | 10(33.3) | 19(16.2) | <0.001^a,c^ | 0.003 | 0.97±1.78 | 0.40±1.54 | 1.17±2.41 | 0.60±1.82 | 0.001^†,a,c^ | 0.198 |
| **Attention/memory** | **43(70.5)** | **50(57.5)** | **23(76.7)** | **73(62.4)** | **0.092** | **0.282** | **3.48±4.56** | **2.76±5.36** | **6.40±8.11** | **3.69±6.35** | **0.005^†,c^** | **0.813** |
| Concentration | 20(32.8) | 7(8.0) | 14(46.7) | 21(17.9) | <0.001^a,c^ | 0.026 | 0.79±1.62 | 0.30±1.23 | 1.70±2.49 | 0.66±1.75 | <0.001^†,a,c^ | 0.633 |
| For.Th.Eve., | 24(39.3) | 48(55.2） | 22(73.3) | 70(59.8) | 0.008^b^ | 0.009 | 1.16±2.19 | 1.46±2.46 | 3.13±3.75 | 1.89±2.92 | 0.003^†,b,c^ | 0.090 |
| Forget to do things | 31(50.4) | 22(25.3) | 11(36.7) | 33(28.2) | 0.006^a^ | 0.003 | 1.52±2.34 | 1.00±2.56 | 1.57±2.96 | 1.15±2.67 | 0.375 | 0.350 |
| **Gastrointestinal** | **58(95.1)** | **73(83.9)** | **26(86.7)** | **99(84.6)** | **0.112** | **0.040** | **9.80±7.32** | **6.24±5.88** | **7.10±7.62** | **6.43±6.35** | **0.006^a^** | **0.002** |
| Dribbling saliva | 30(49.2) | 37(42.5) | 14(46.7) | 51(43.6) | 0.719 | 0.477 | 2.16±3.22 | 1.56±2.70 | 2.53±3.98 | 1.81±3.09 | 0.501^†^ | 0.478 |
| Swallowing | 50(82.0) | 29(33.3) | 17(56.7) | 46(39.3) | <0.001^a,b,c^ | <0.001 | 2.79±2.87 | 0.76±1.58 | 2.40±3.60 | 1.18±2.37 | <0.001^†,a,c^ | <0.001 |
| Constipation | 51(83.6) | 61(70.1) | 18(60) | 79(67.5) | 0.040^b^ | 0.022 | 4.85±4.22 | 3.87±4.07 | 2.17±3.40 | 3.44±3.97 | 0.006^†,b^ | 0.028 |
| **Urinary** | **56(91.8)** | **54(62.1)** | **17(56.7)** | **71(60.7)** | **<0.001^a,b^** | **<0.001** | **13.74±11.07** | **5.20±7.62** | **5.30±7.11** | **5.22±7.46** | **<0.001^†,a,b^** | **<0.001** |
| Urinary urgency | 46(75.4) | 30(34.5) | 13(43.3) | 43(36.8) | <0.001^a,b^ | <0.001 | 4.75±4.35 | 1.52±2.71 | 2.00±3.14 | 1.64±2.82 | <0.001^†,a,b^ | <0.001 |
| Urinary frequency | 44(72.1) | 34(39.1) | 11(36.7) | 45(38.5) | <0.001^a,b^ | <0.001 | 4.31±3.95 | 1.61±2.70 | 1.37±2.36 | 1.55±2.61 | <0.001^†,a,b^ | <0.001 |
| Nocturia | 45(73.8) | 41(47.1) | 13(43.3) | 54(46.2) | <0.001^a,b^ | <0.001 | 4.67±4.11 | 2.07±3.13 | 1.93±3.32 | 2.03±3.14 | <0.001^†,a,b^ | <0.001 |
| **Sexual dysfunction** | **54(88.5)** | **14(16.1)** | **11(36.7)** | **25(21.4)** | **<0.001^a,b,c^** | **<0.001** | **11.89±8.96** | **2.01±5.55** | **3.47±6.14** | **2.38±5.72** | **<0.001^†,a,b^** | **<0.001** |
| Los. Sex. Int., | 49(80.3) | 13(14.9) | 9(30) | 22(18.8) | <0.001^a,b^ | <0.001 | 5.41±4.65 | 0.93±2.75 | 1.70±3.26 | 1.13±2.89 | <0.001^†,a,b^ | <0.001 |
| Pro.Hav.S. | 50(82.0) | 14(15.6) | 9(30) | 23(19.7) | <0.001^a,b^ | <0.001 | 6.48±5.05 | 1.08±2.93 | 1.77±3.33 | 1.26±3.04 | <0.001^†,a,b^ | <0.001 |
| **Miscellaneous** | **48(78.7)** | **67(77.0)** | **23(76.7)** | **90(76.9)** | **0.964** | **0.789** | **6.11±6.94** | **6.72±6.66** | **5.00±5.23** | **6.28±6.35** | **0.457** | **0.872** |
| Pain | 25(41.0) | 30(34.5) | 11(36.7) | 41(35.0) | 0.722 | 0.436 | 1.89±3.27 | 1.37±2.81 | 1.80±3.17 | 1.48±2.90 | 0.558 | 0.397 |
| Hyposmia | 22(36.1) | 41(47.1) | 8(26.7) | 49(41.9) | 0.108 | 0.452 | 1.36±2.56 | 2.71±3.88 | 0.93±2.07 | 2.26±3.58 | 0.031^†,c^ | 0.391 |
| Weight change | 17(27.9) | 9(10.3) | 4(13.3) | 13(11.1) | 0.017^a^ | 0.005 | 0.97±2.59 | 0.54±1.84 | 0.67±2.17 | 0.57±1.92 | 0.499 | 0.251 |
| Hyperhydrosis | 26(42.6) | 36(41.4) | 12(40) | 48(41.0) | 0.971 | 0.837 | 1.90±2.83 | 2.10±3.26 | 1.60±2.79 | 1.97±3.14 | 0.729 | 0.880 |

*p*1 and *p*2 are t the *p*-values for the comparison of the frequency and scores of non-motor symptoms among the three groups of MSA, PD, and PSP, respectively. *p*c1 and *p*c2 are the p-values for the comparison of the frequency and scores of non-motor symptoms in the MSA group and the PD+PSP group, respectively. Comparisons of frequency of non-motor symptom were performed using the chi-square test. Unless otherwise specified, comparisons of continuous variables among three groups were performed using SPSS-analysis of variance. ^†^ Kruskal-Wallis test was used to detect group differences. ^a^ significant differences between MSA and PD; ^b^ significant differences between MSA and PSP; ^c^ significant differences between PD and PSP. Values in bold represent statistics for total NMSS symptoms or subdomain symptoms between groups, whereas the rest values correspond to each individual symptom included in a subdomain.

Abbreviations: NMS, non-motor symptoms; NMSS, the Non-Motor Symptom Scale; MSA, multiple system atrophy; PD, Parkinson’s disease; PSP, progressive supranuclear palsy; Ort.sym., Orthostatic symptoms; F.B.F., Falls because of fainting; D.S., Daytime sleepiness; Dif.F.Asl., Difficulty falling asleep; L. I. S., Lost interest in surroundings; Dif.Ex.Plea., Difficulty experiencing pleasure; P./H., Perceptual/hallucinations; For.Th.Eve., Forget things or events; Los. Sex. Int., loss of sexual interest; Pro.Hav.S., Problems having sex

**Supplementary Table 2 | Correlations of non-motor symptoms with other clinical features in MSA, PD and PSP**

|  | **Total NMSS** | | **Cardiovascular** | **Sleep** | **Mood** | **Perceptual problems** | **Memory** | **Gastrointestinal** | **Urinary** | **Sexual** | **Miscellaneous** |
| --- | --- | --- | --- | --- | --- | --- | --- | --- | --- | --- | --- |
| **MSA** |  |  | |  |  |  |  |  |  |  |  |
| Age | 0.058 | | -0.006 | 0.069 | 0.126 | 0.022 | -0.004 | 0.139 | 0.050 | -0.114 | 0.066 |
| Gender | 0.130 | | -0.025 | -0.012 | 0.031 | 0.100 | 0.089 | 0.035 | 0.184 | **0.466^*^** | -0.017 |
| Duration | 0.161 | | 0.017 | 0.056 | 0.072 | 0.185 | 0.015 | 0.321 | 0.190 | -0.008 | 0.143 |
| BMI | 0.131 | | 0.292 | 0.013 | 0.007 | 0.149 | 0.038 | 0.118 | 0.129 | 0.213 | 0.058 |
| Education | -0.011 | | -0.188 | -0.035 | 0.004 | 0.071 | -0.127 | -0.073 | -0.154 | 0.233 | 0.039 |
| UMSARS-IV | 0.180 | | 0.143 | 0.195 | 0.193 | 0.189 | 0.136 | 0.183 | 0.148 | -0.073 | 0.234 |
| UMSARS-II | 0.245 | | 0.074 | 0.319 | 0.134 | 0.094 | 0.101 | 0.416 | 0.078 | -0.134 | **0.456^*^** |
| **PD** |  | |  |  |  |  |  |  |  |  |  |
| Age | 0.081 | | 0.128 | -0.171 | -0.153 | -0.029 | 0.122 | 0.295 | 0.259 | -0.237 | 0.054 |
| Gender | 0.088 | | <0001 | -1.191 | -0.144 | 0.066 | 0.054 | 0.158 | 0.244 | 0.227 | -0.153 |
| Duration | 0.252 | | 0.052 | 0.234 | -0.017 | 0.139 | 0.001 | 0.128 | 0.196 | 0.078 | 0.099 |
| BMI | -0.042 | | -0.052 | -0.049 | -0.114 | 0.129 | 0.062 | -0.123 | -0.253 | 0.098 | -0.018 |
| Education | 0.048 | | -0.011 | -0.027 | -0.036 | 0.023 | -0.059 | -0.041 | 0.028 | 0.069 | 0.023 |
| H&Y stage | **0.515^*^** | | 0.156 | **0.414^*^** | 0.339 | 0.179 | 0.258 | **0.354^*^** | 0.274 | 0.064 | 0.253 |
| UPDRS-III | **0.430^*^** | | 0.136 | **0.371^*^** | **0.410^*^** | 0.237 | 0.208 | **0.392^*^** | 0.153 | 0.070 | 0.239 |
|  |  | |  |  |  |  |  |  |  |  |  |
| **PSP** |  | |  |  |  |  |  |  |  |  |  |
| Age | <0.001 | | -0.133 | -0.182 | -0.175 | -0.233 | -0.027 | 0.134 | 0.011 | -0.283 | 0.002 |
| Gender | -0.140 | | 0.017 | -0.167 | -0.238 | 0.189 | -0.020 | -0.210 | 0.138 | 0.504 | -0.141 |
| Duration | 0.098 | | 0.122 | -0.204 | -0.106 | 0.213 | 0.099 | -0.044 | 0.267 | -0.274 | 0.265 |
| BMI | -0.040 | | 0.027 | -0.024 | -0.159 | 0.154 | -0.188 | 0.024 | 0.055 | 0.274 | 0.249 |
| Education | -0.033 | | 0.128 | -0.377 | -0.287 | 0.052 | -0.101 | -0.100 | 0.270 | 0.333 | 0.157 |
| H&Y stage | 0.084 | | 0.048 | -0.005 | 0.142 | -0.019 | -0.041 | 0.038 | -0.084 | -0.415 | -0.069 |
| UPDRS-III | -0.017 | | -0.014 | -0.081 | 0.161 | 0.110 | 0.005 | 0.184 | -0.088 | -0.359 | -0.009 |

Abbreviations: MSA, multiple system atrophy; PD, Parkinson’s disease; PSP, progressive supranuclear palsy. UMSARS-IV, the Unified Multiple System Atrophy Rating Scale part IV; UMSARS-II, the Unified Multiple System Atrophy Rating Scale part II; H&Y stage, Hoehn & Yahr stage; UPDRS-III, Movement Disorders Society–revised Unified Parkinson’s Disease Rating Scale part III; NMSS, the Non-Motor Symptom Scale. Values in bold indicate statistically significant correlations after Bonferroni’s correction (* *p* < 0.00083).

**Supplementary Table 3 | The NMS number distribution in MSA, PD and PSP**

| Number of non-motor symptoms | MSA | | PD | | PSP | |
| --- | --- | --- | --- | --- | --- | --- |
|  | proportion（%） | Cumulative percentage（%） | proportion（%） | Cumulative percentage（%） | proportion | Cumulative percentage（%） |
| 0 | 0 | 0 | 1.1 | 1.1 | 0 | 0 |
| 1-5 | 1.6 | 1.6 | 25.3 | 26.4 | 23.3 | 23.3 |
| 6-10 | 13.1 | 14.8 | 44.8 | 71.3 | 43.3 | 66.7 |
| 11-15 | 18.0 | 32.8 | 18.4 | 89.3 | 13.3 | 80.0 |
| 16-20 | 42.6 | 75.4 | 8.0 | 97.7 | 6.7 | 86.7 |
| 21-25 | 19.7 | 95.1 | 2.3 | 100 | 10.0 | 96.7 |
| 26-30 | 4.9 | 100 | 0 | 100 | 3.3 | 100 |

Abbreviations: NMS, non-motor symptoms; MSA, multiple system atrophy; PD, Parkinson’s disease; PSP, progressive supranuclear palsy.

**Supplementary Table 4 | The timing of NMS onset relative to motor symptoms onset in MSA**

| Symptoms | Number of patients (n，%) | Time from the motor symptoms onset, years (mean±SD) | Time from the motor symptoms onset, years, (median (95% CI)) |
| --- | --- | --- | --- |
| RBD | 50(81.9) | -2.81±4.51 | -1.00(-7.00, 0.20) |
| Constipation | 51(83.6) | -1.54±6.32 | 0.45(-2.75, 2.00) |
| Problems having sex | 50(82.0) | -1.35±4.70 | 0.00(-2.00, 1.00) |
| loss of sexual interest | 49(80.3) | -0.45±3.61 | 0.00(-1.00, 2.00) |
| Concentration | 20(32.8) | -0.05±9.50 | 2.00(1.25, 3.75) |
| Motor symptoms | 65(100) | 0 | 0 |
| Urinary frequency | 44(72.1) | 0.22±2.97 | 1.00(-1.00, 2.00) |
| Urinary urgency | 46(75.4) | 0.24±2.74 | 0.85(-1.00, 2.00) |
| Orthostatic symptoms | 42(68.9) | 0.33±3.78 | 0.85(0.00, 1.95) |
| Hyperhydrosis | 26(42.6) | 0.45±3.02 | 1.00(-1.25, 2.63) |
| Restless legs | 33(54.1) | 0.66±2.54 | 1.20(-0.23, 2.15) |
| Nocturia | 45(73.8) | 0.67±2.64 | 1.00(0.00, 2.00) |
| Urinary incontinence | 31(50.0) | 0.98±1.45 | 1.00(0.00, 2.13) |
| Weight change | 17(27.9) | 1.18±1.88 | 1.50(0.50, 2.00) |
| Difficulty falling asleep | 39(63.9) | 1.27±2.20 | 1.00(0.00, 2.50) |
| Hyposmia | 22(36.1) | 1.40±4.46 | 2.00(1.00, 4.00) |
| Fatigue | 46(75.4) | 1.55±1.92 | 1.80(1.00, 2.00) |
| Dribbling saliva | 30(49.2) | 1.60±5.70 | 1.95(1.13, 4.00) |
| Depression | 45(73.8) | 1.63±1.53 | 1.80(0.55, 2.43) |
| Falls because of fainting | 32(52.5) | 1.71±1.64 | 1.30(0.13, 3.00) |
| Anxiety | 36(59.0) | 1.72±1.37 | 1.80(1.00, 2.25) |
| Pain | 25(41.0) | 1.79±1.96 | 1.85(1.00, 2.68) |
| Hallucinations | 7(11.5） | 1.85±1.01 | 2.00(1.28, 2.55) |
| Lack motivation | 50(82.0) | 1.86±1.29 | 2.00(1.00, 2.70) |
| Delusions | 7(11.5) | 2.00±1.22 | 2.00(1.00, 3.00) |
| Forget things or events | 24(39.3) | 2.00±1.51 | 2.00(1.00, 3.00) |
| Flat mood | 33(54.1) | 2.02±1.33 | 2.00(1.00, 3.00) |
| Difficulty experiencing pleasure | 38(62.3) | 2.04±1.59 | 1.80(1.00, 3.10) |
| Daytime sleepiness | 30(49.2) | 2.05±1.94 | 2.00(1.00, 2.95) |
| Lost interest in surroundings | 41(67.2) | 2.06±1.49 | 2.00(1.00, 3.00) |
| Forget to do things | 31(50.4) | 2.09±1.81 | 1.80(1.00, 4.00) |
| Double vision | 22(36.1) | 2.14±2.15 | 2.00(1.00, 3.50) |
| Swallowing | 50(82.0) | 2.34±1.91 | 2.10(1.03, 3.73) |

Abbreviations: MSA, multiple system atrophy; RBD, REM Sleep Behavior Disorder.
